# Supplementary material for: Division rate, cell size and proteome allocation: impact on gene expression noise and implications for the dynamics of genetic circuits
Source: R Soc Open Sci. 2018 Mar 21;5(3):172234. doi: 10.1098/rsos.172234 (PMC5882738; doi:10.1098/rsos.172234)
Supplement: Supplemental Figures [file rsos172234supp1.pdf]

Supplemental Figures

Division rate, cell size and proteome allocation: impact on gene expression noise and implications for the dynamics of genetic circuits

François Bertaux[1,2,3], Samuel Marguerat[2,3,4], Vahid Shahrezaei[1,4]

1. Department of Mathematics, Imperial College London, London SW7 2AZ, UK
2. MRC London Institute of Medical Sciences (LMS), London W12 0NN, UK
3. Institute of Clinical Sciences (ICS), Faculty of Medicine, Imperial College London, London, W12 0NN, UK
4. To whom correspondence should be addressed

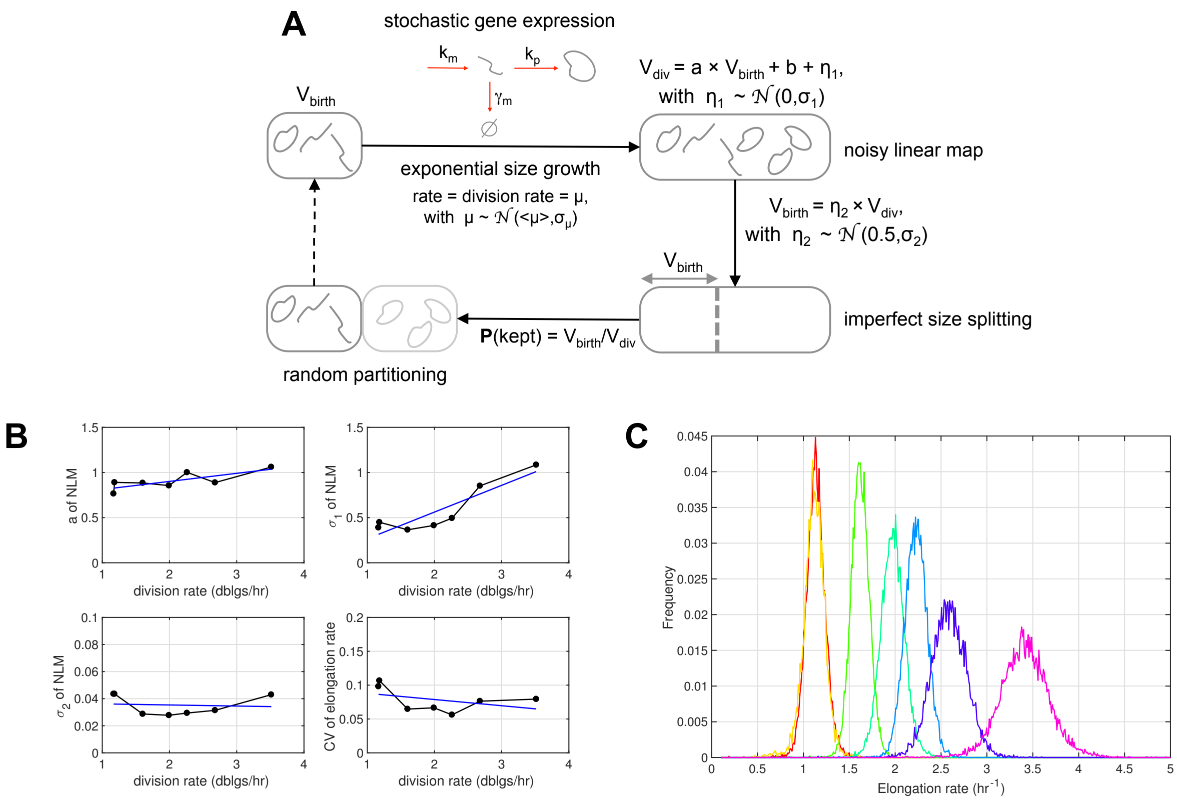

Figure S1: *Realistic modeling of cell size noise and cell growth rate noise as a function of division rates.* **(A)** Model description. The model is as described in Figure 2-A, with the following additional assumptions. The exponential cell growth rate is assumed to be normally distributed around the population average. Noise and memory in cell division and birth size are modeled with the recently proposed 'noisy linear map' (Tanouchi *et al*, 2015; Jun & Taheri-Araghi, 2015). The case $a = 1$ corresponds to the 'adder' principle (Taheri-Araghi *et al*, 2015) and $a = 0$ means that birth size is independent of previous birth size ('sizer'). **(B)** Extraction of noisy linear map and cell growth rate noise parameters as a function of population division rate from recent mother machine data at different growth conditions (Taheri-Araghi *et al*, 2015). We fit simple linear trends to the extracted data to extrapolate to intermediate growth conditions. $b(\mu)$ is chosen such that average birth size matches $V_{birth}(\mu) = \frac{b(\mu)}{2-a(\mu)} = 0.19 \times 2^{1.11\times\mu}$. This exponential dependency has been shown in (Taheri-Araghi *et al*, 2015) to describe the data very well (cf Figure S1). **(C)** Histograms of individual cell growth rates at different growth conditions show that using normal distributions is accurate.

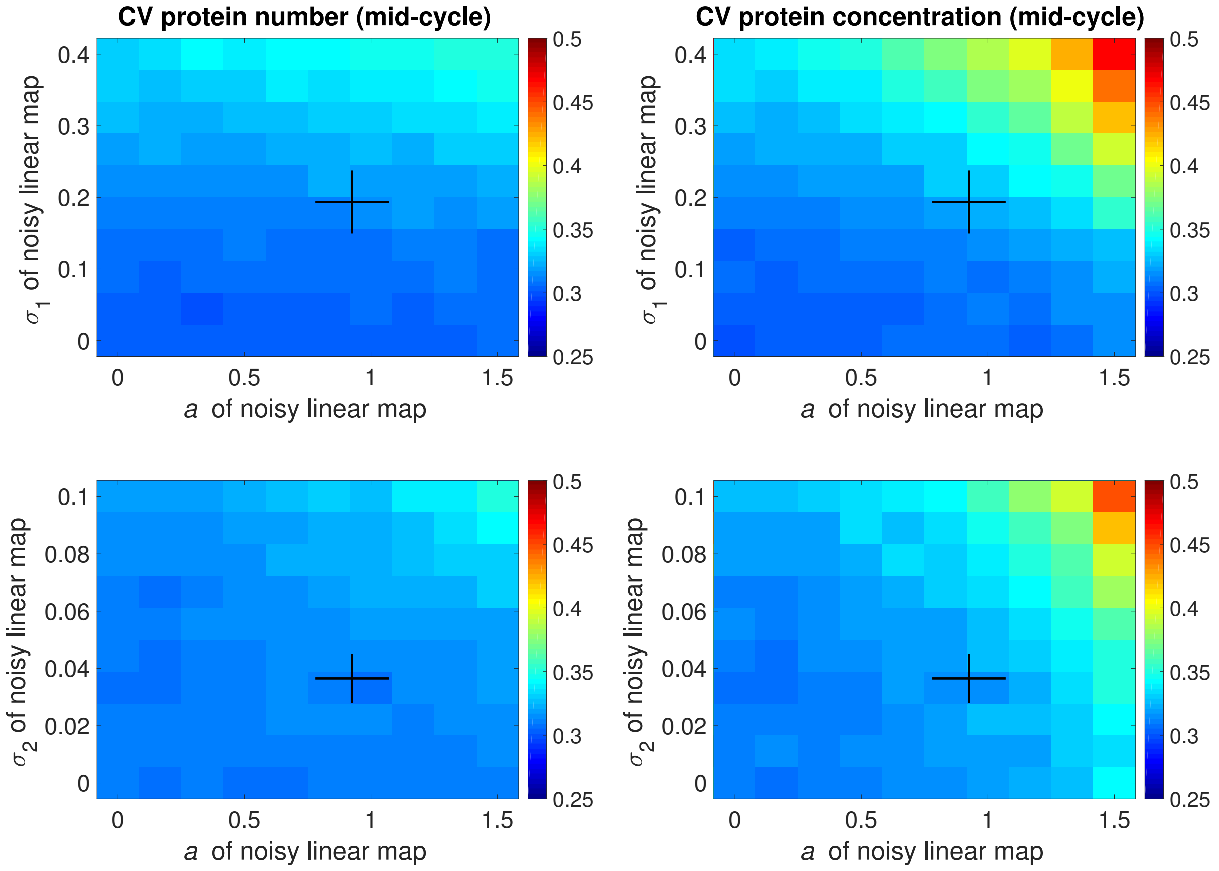

Figure S2: *Protein concentration noise at the middle of the cell cycle.* Same as Figure 2-C except that the concentration noise is computed for cells at the middle of their cell cycle.

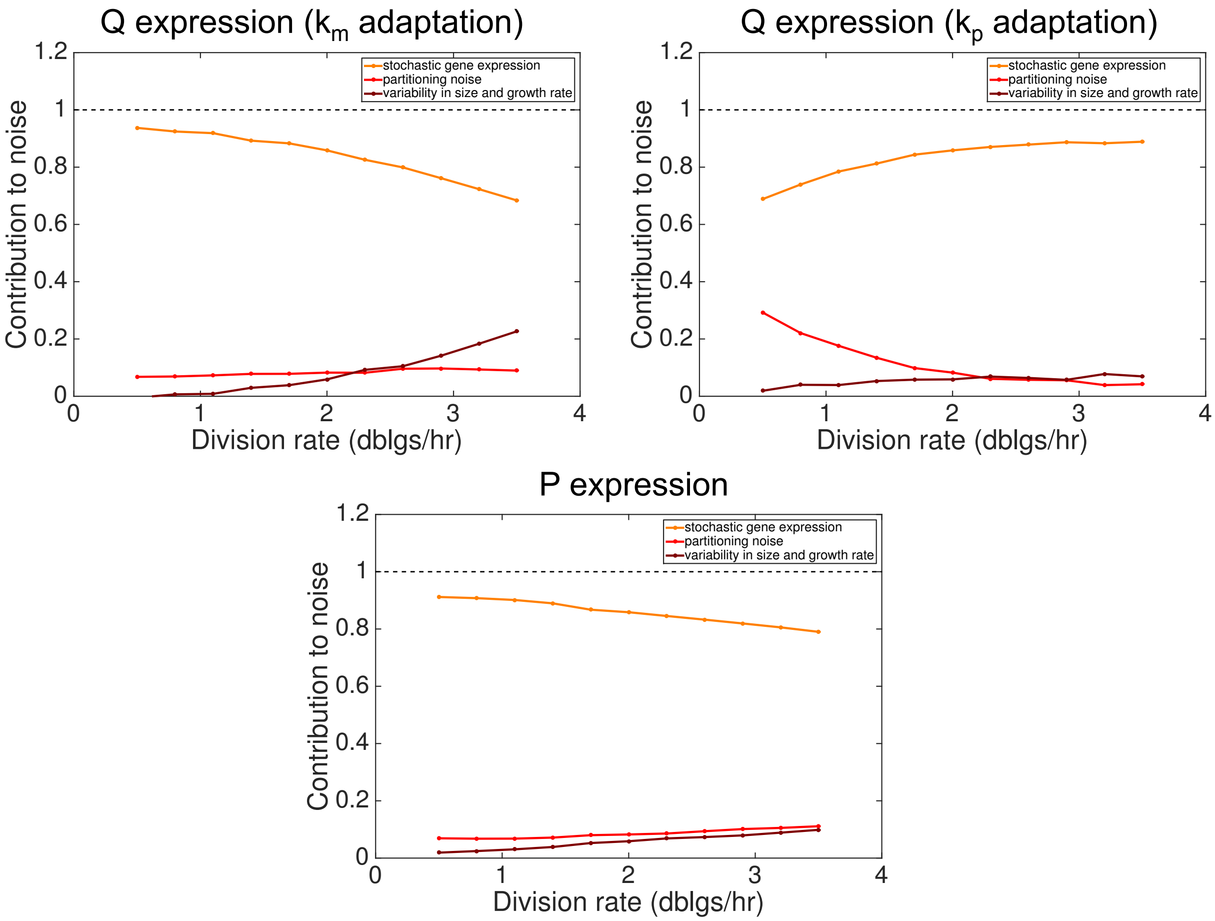

Figure S3: *Distinct contributions to protein concentration noise at cell birth.* The simulation data from Figure 3 and Figure 4 is used to compute the relative contribution of three noise sources: stochastic gene expression, partitioning noise, and variability in division size and growth rate.

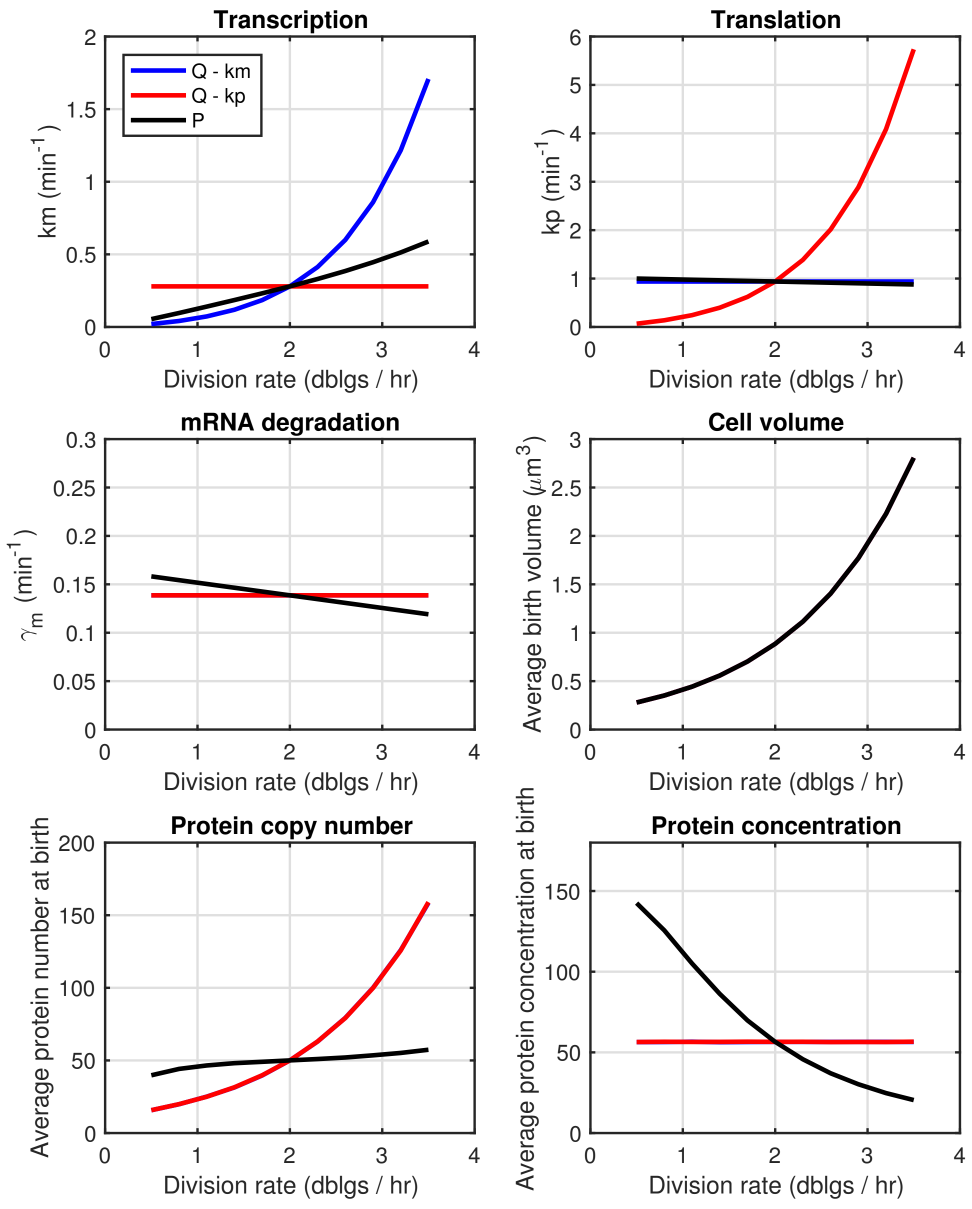

Figure S4: *Growth rate dependence of gene expression parameters for Q (transcriptional or translational adjustment) and P (constitutive expression).* Related to Figure 3 (*Q-km* and *Q-kp*) and Figure 4 (P) of main text. The same cell volume dependency with the division rate is used in all cases ($V_{birth}(\mu) = 0.19 \times 2^{1.11\times\mu}$, Taheri-Araghi *et al* (2015)). For P, to obtain relative changes of k_m, k_p and γ_m with the division rate, data was extracted from Figure 1 in (Klumpp *et al*, 2009), which corresponds to constitutively expressed promoters, and simple analytic functions were fitted to the data to allow extrapolation to intermediate division rates.

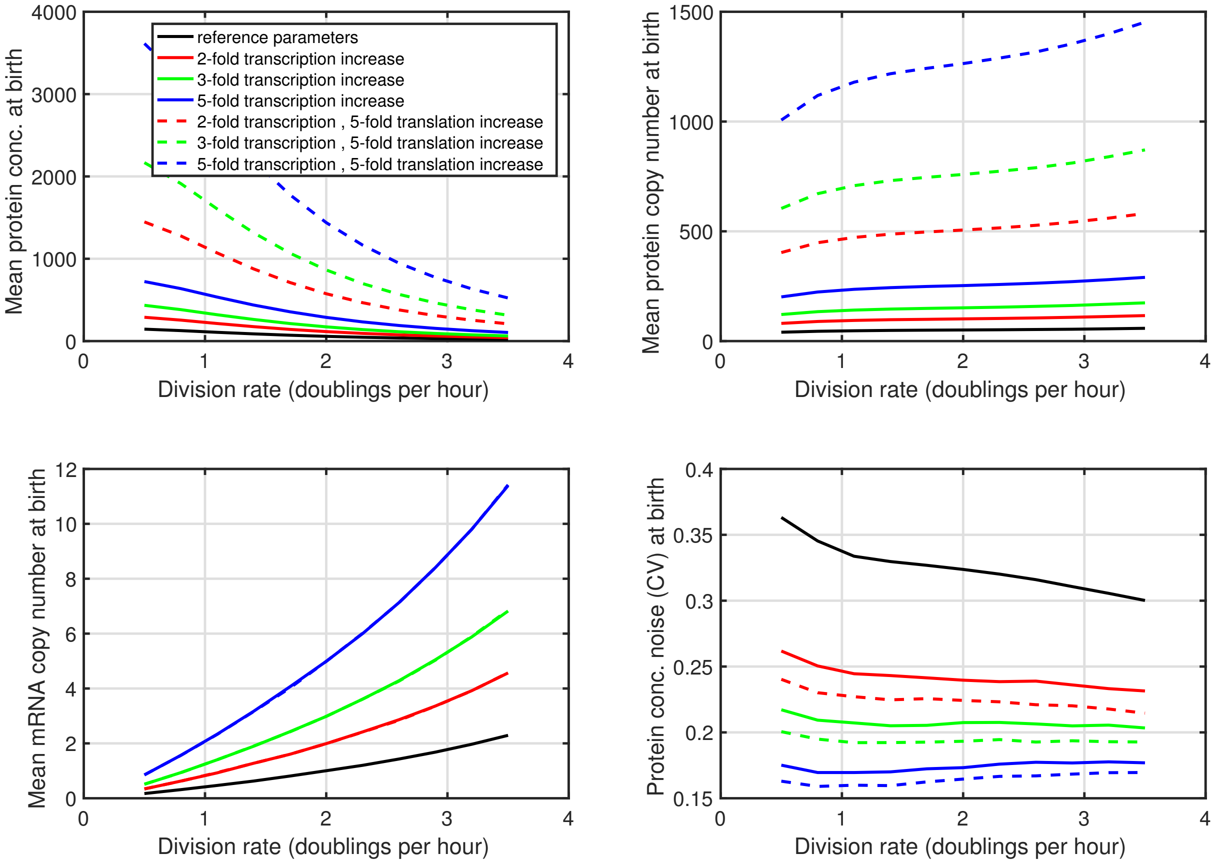

Figure S5: *Impact of baseline expression level on protein concentration noise dependency with the division rate for P proteins.* The analysis of Figure 4 is repeated for various baseline expression levels, obtained by scaling up the reference transcription rate, translation rate, or both. As transcription rate increases, intrinsic noise decreases, and the protein concentration noise decreases. The translation rate has a lower impact than transcription rate as mRNA noise dominates.

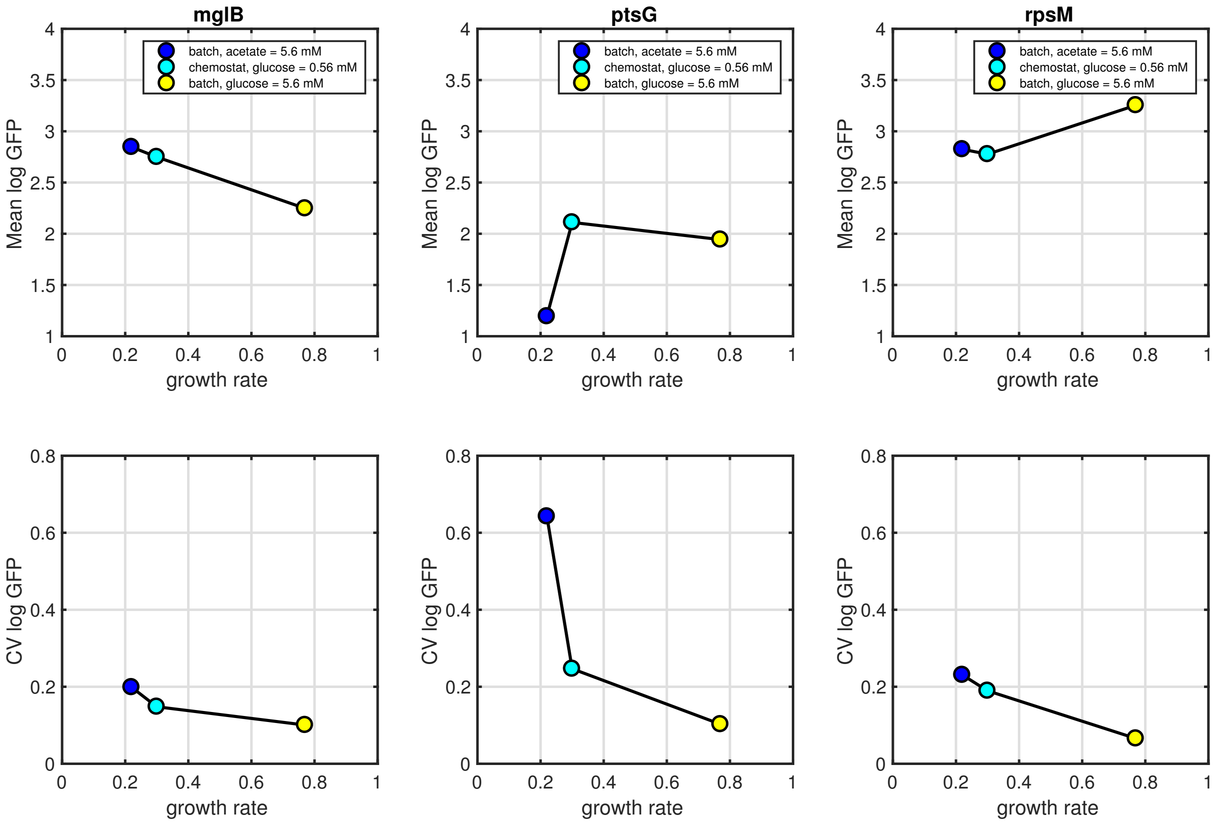

Figure S6: *Single-cell expression data at different growth rates.* Average protein expression and noise for three promoter-GFP fusions constructs at different growth rates. Data from (Nikolic *et al*, 2013) (as growth rates for batch cultures were not reported, we used values for matching media and background strain from (Thakur *et al*, 2010)). Contribution of cell-to-cell variability in size within each condition was reduced by stringent gating. mglB and ptsG are transporters, and rpsM is a ribosomal protein. Interestingly, consistently with our modeling predictions presented in Figure 4, for the mglB promoter noise decreases with growth rate despite a decrease in average expression. Average expression of ptsG and rpsM increases with the growth rate, and noise also decreases, as trivially expected for R-like growth rate dependency.

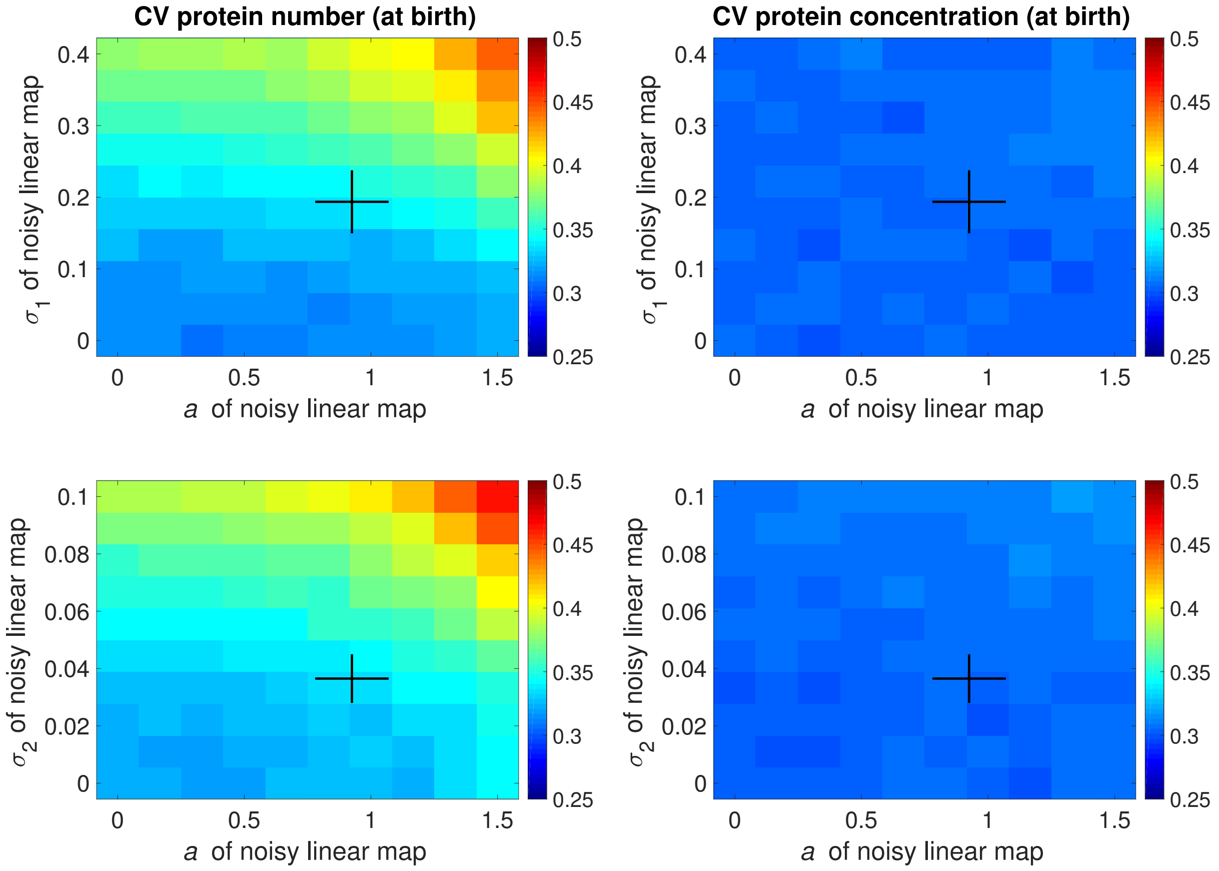

Figure S7: *Protein concentration noise is robust to variability in size when the transcription rate scales with cell size.* Same as Figure 2-C, except that transcription rate is proportional to size instead of being constant. The proportionality constant has been chosen to obtain the same average number of protein at birth.
