# Supplementary figures and images for: Division rate, cell size and proteome allocation: impact on gene expression noise and implications for the dynamics of genetic circuits
Source: R Soc Open Sci. 2018 Mar 21;5(3):172234. doi: 10.1098/rsos.172234 (PMC5882738; doi:10.1098/rsos.172234)

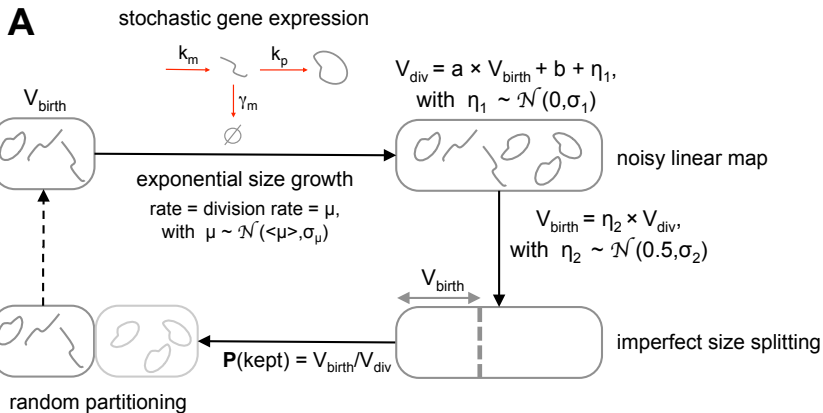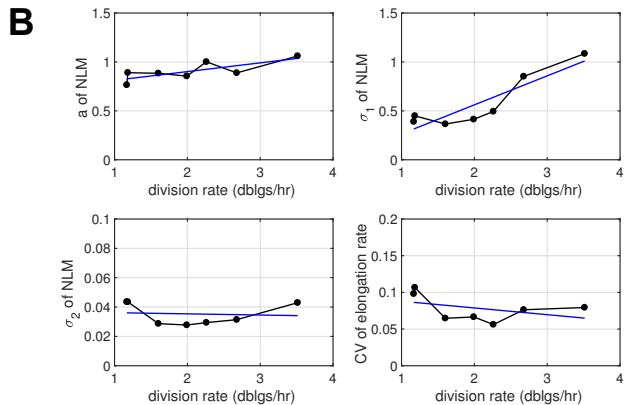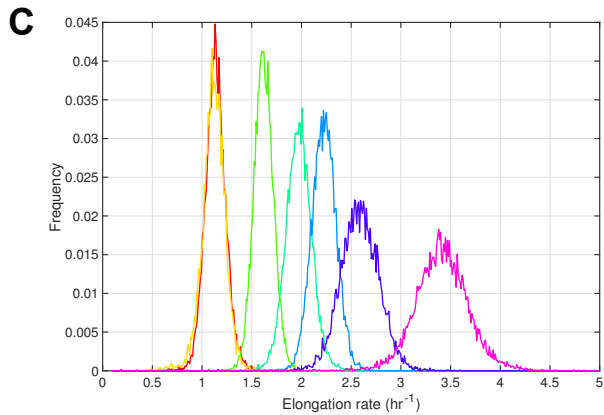

Supplement: Supplemental Figure 1: Realistic modeling of cell size noise and cell growth rate noise as a function of division rates [file rsos172234supp2.pdf]

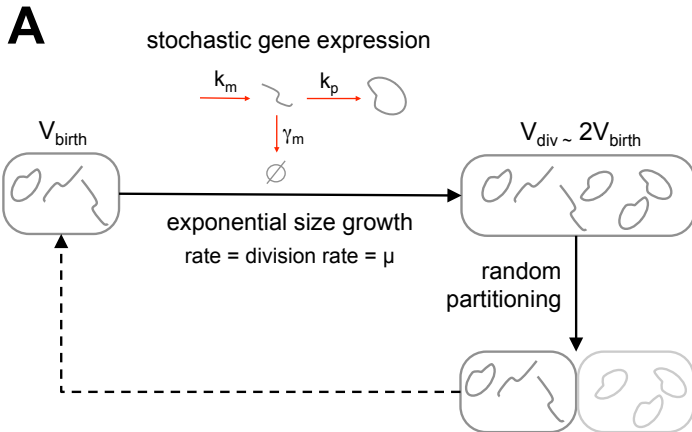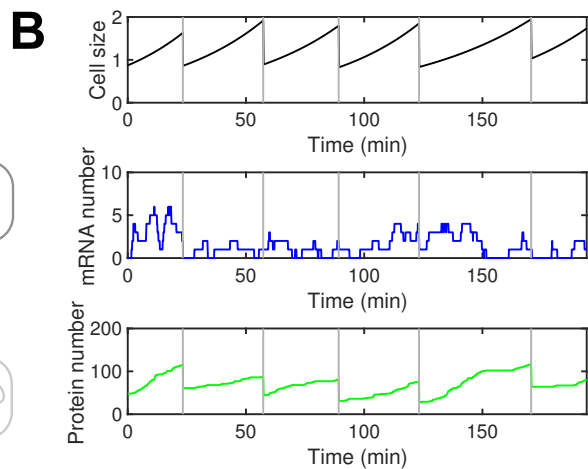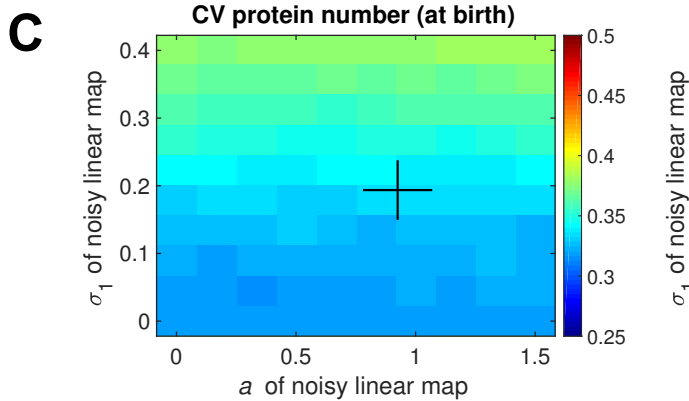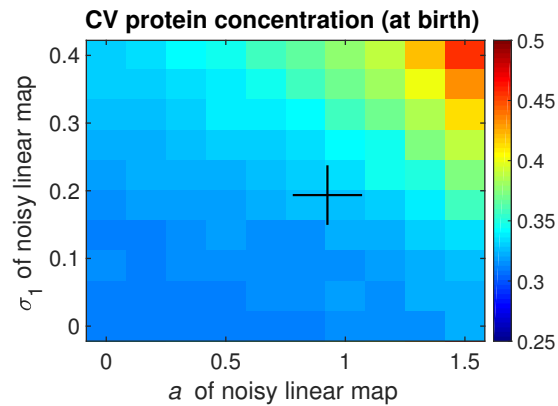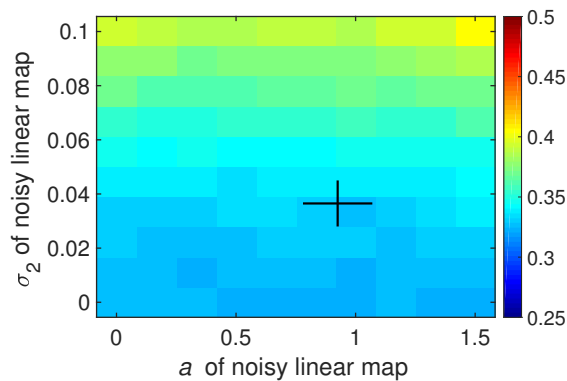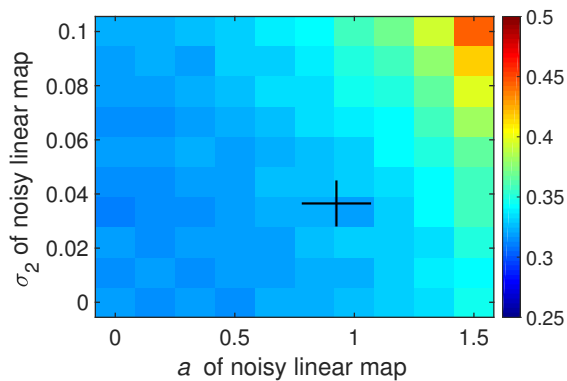

Supplement: Supplemental Figure 2: Protein concentration noise at the middle of the cell cycle [file rsos172234supp3.pdf]

### Q expression ( $k_m$ adaptation)

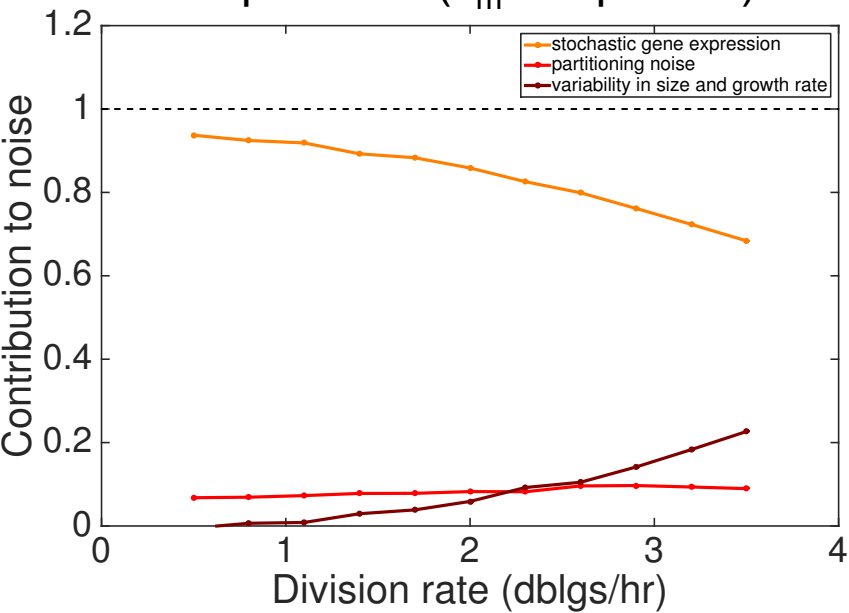

### Q expression ( $k_p$ adaptation)

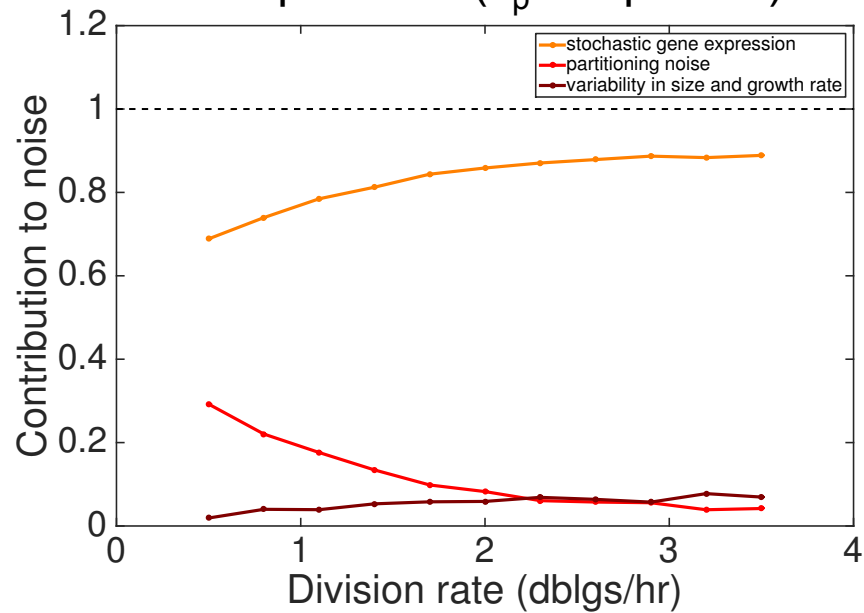

### P expression

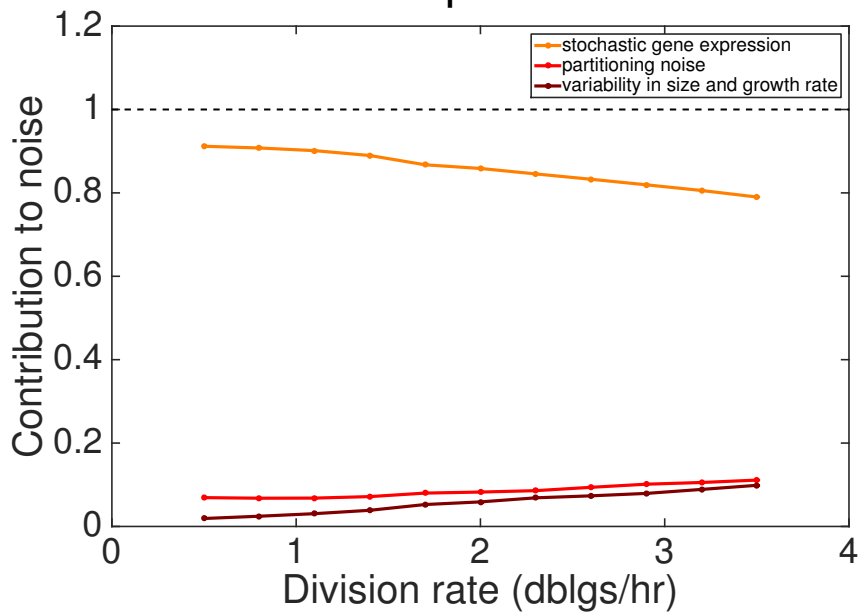

Supplement: Supplemental Figure 3: Distinct contributions to protein concentration noise at cell birth [file rsos172234supp4.pdf]

Mean protein  
concentration

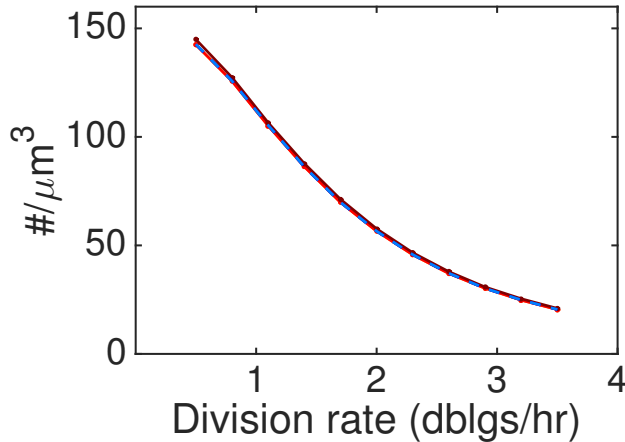

Mean mRNA #

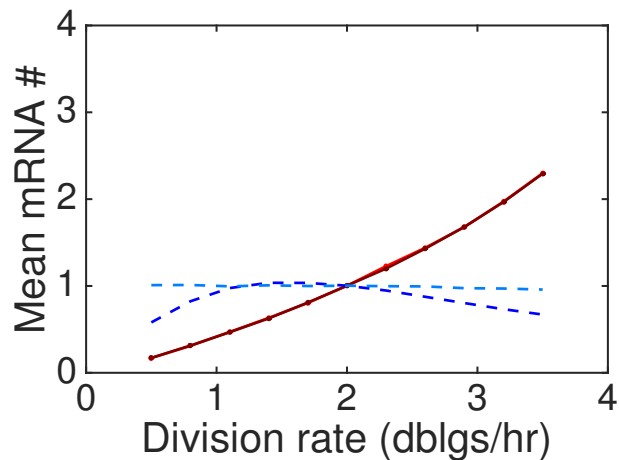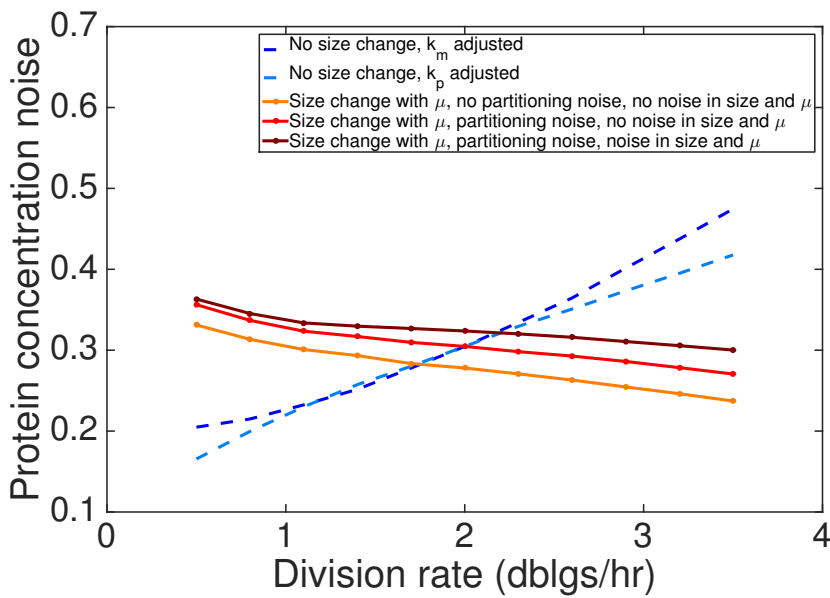

Supplement: Supplemental Figure 4: Growth rate dependence of gene expression parameters for Q (transcriptional or translational adjustment) and P (constitutive expression). [file rsos172234supp5.pdf]

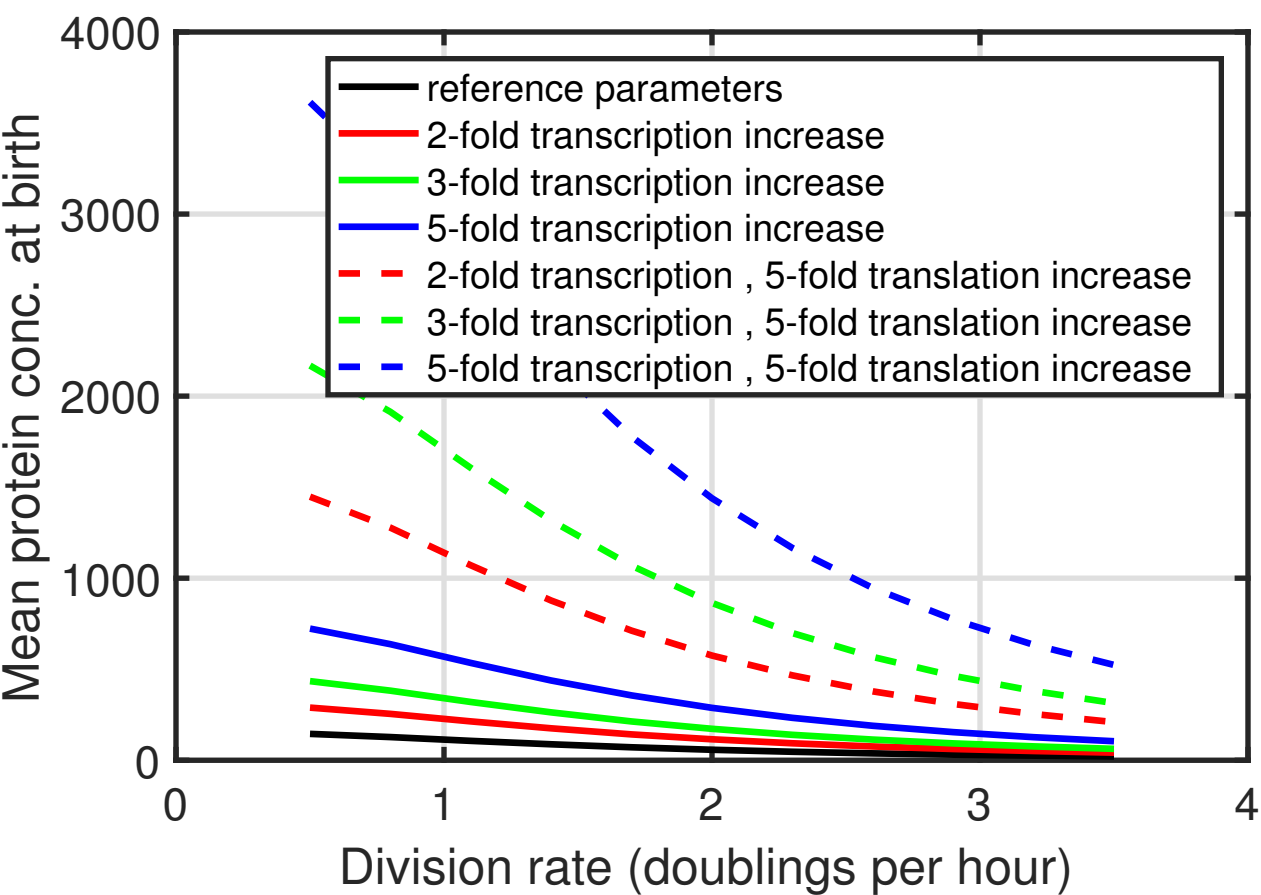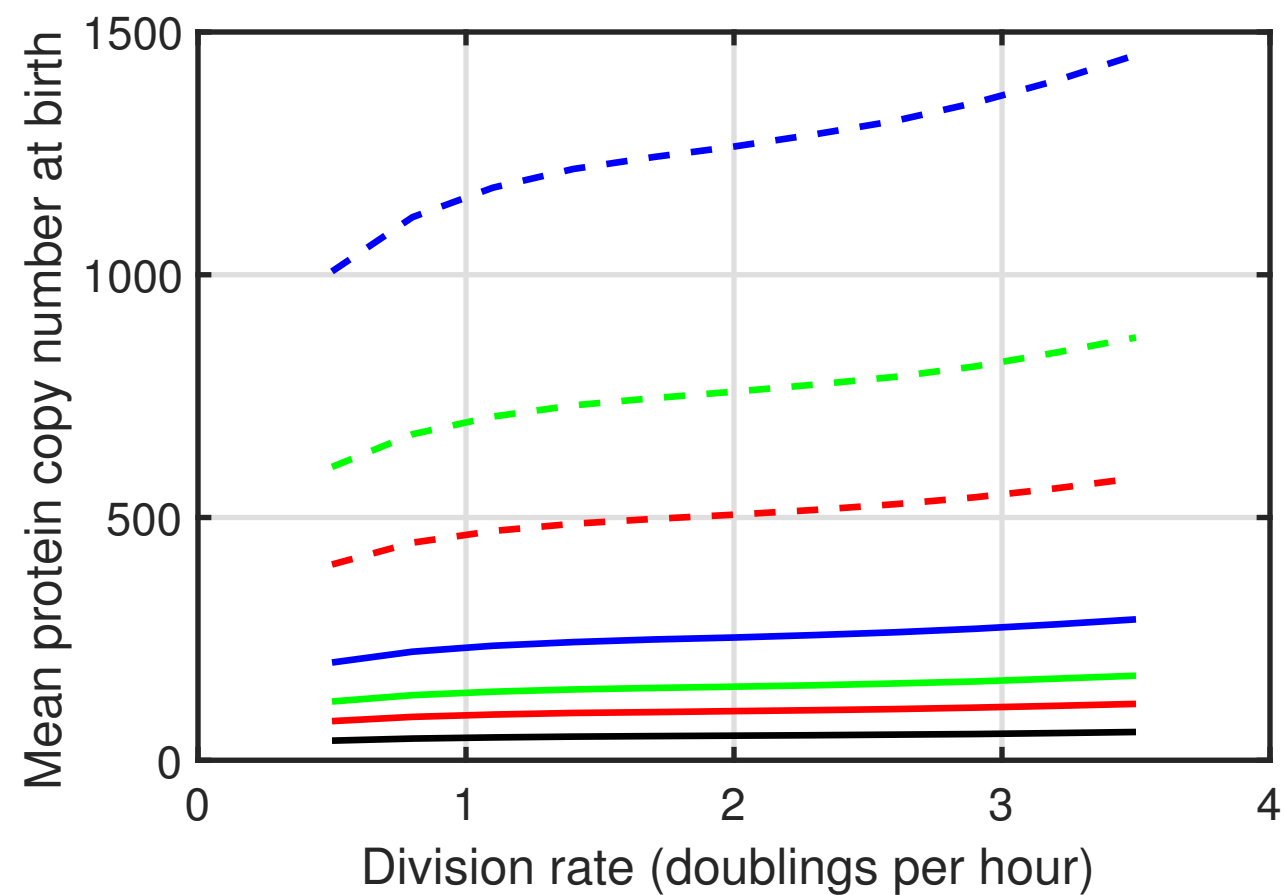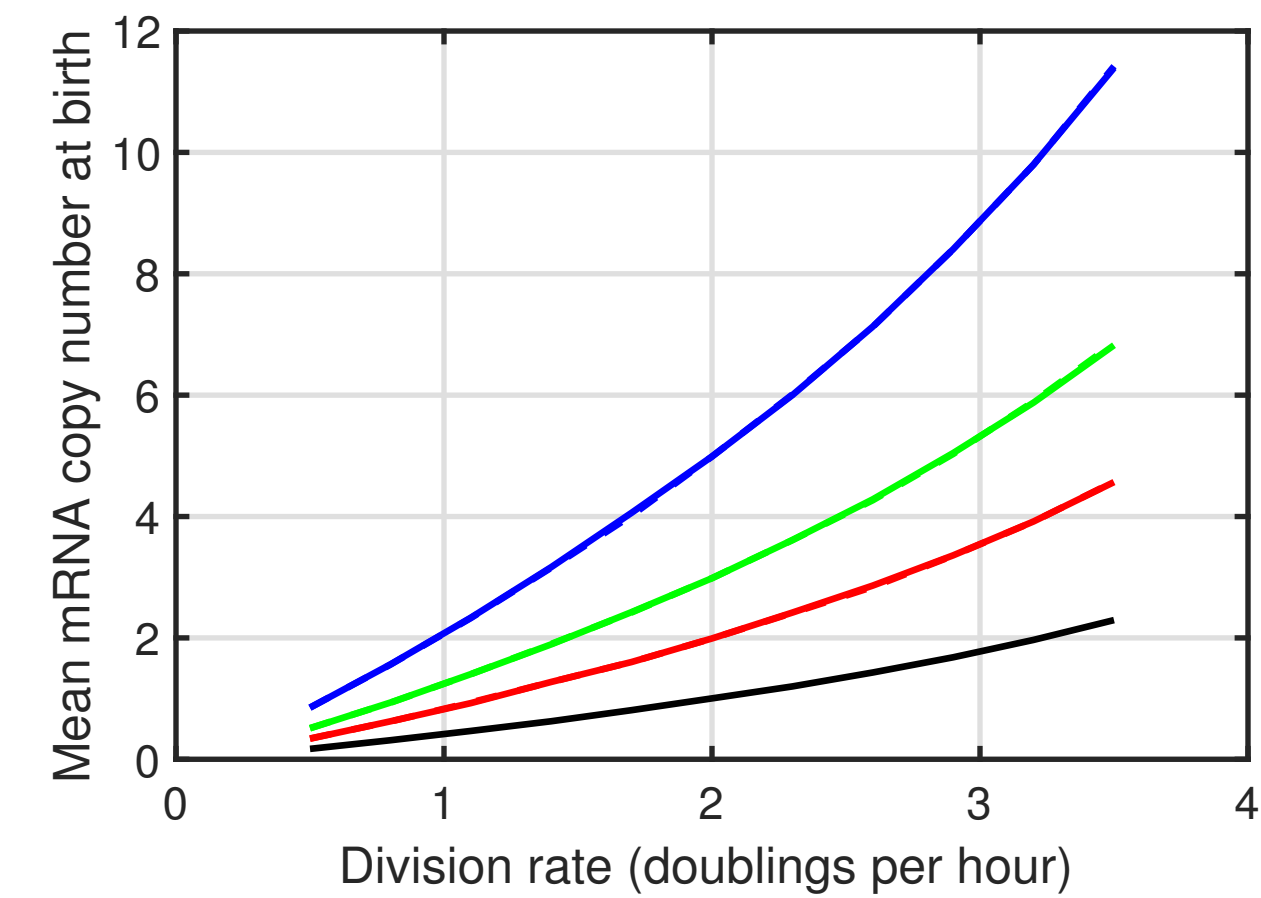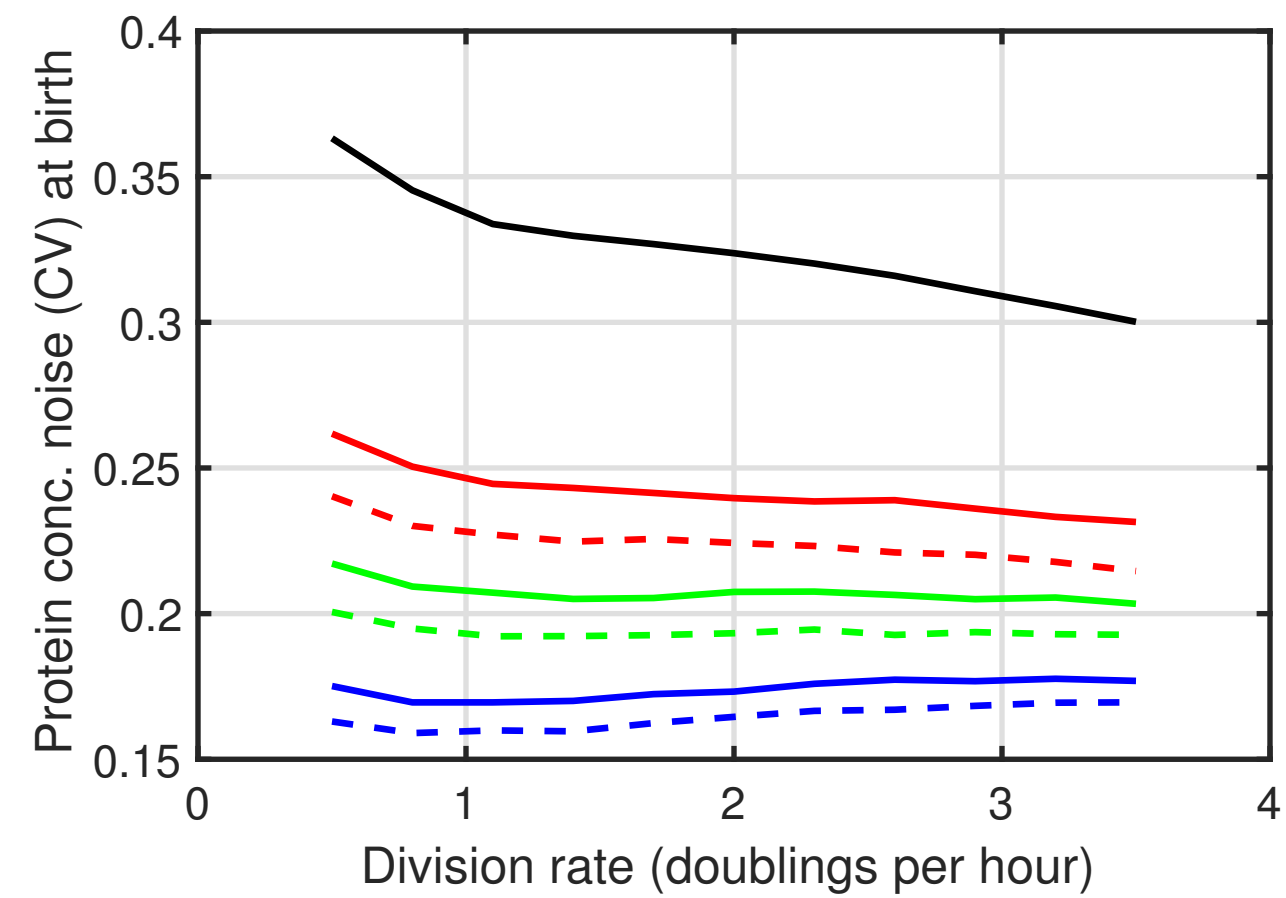

Supplement: Supplemental Figure 5: Impact of baseline expression level on protein concentration noise dependency with the division rate for P proteins [file rsos172234supp6.pdf]

*mglB*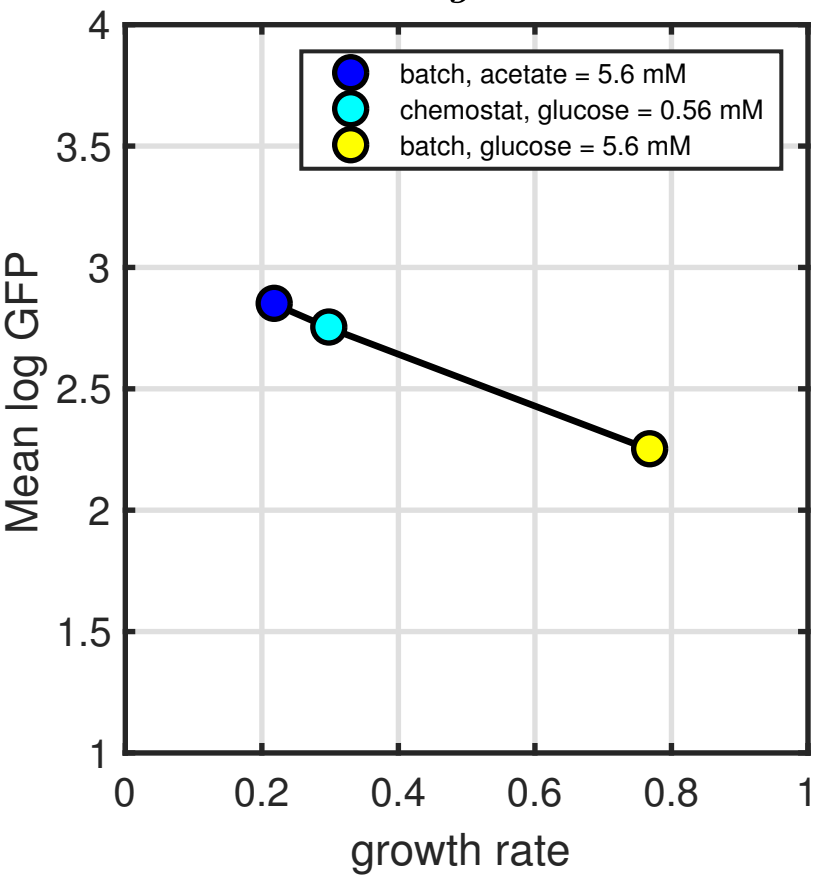*ptsG*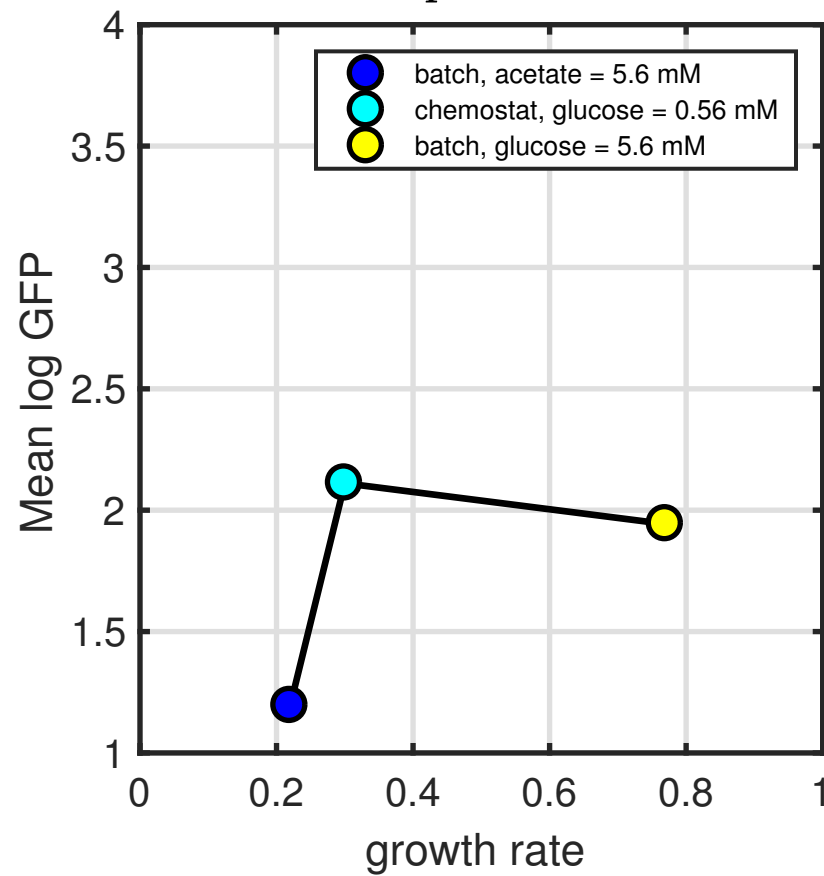*rpsM*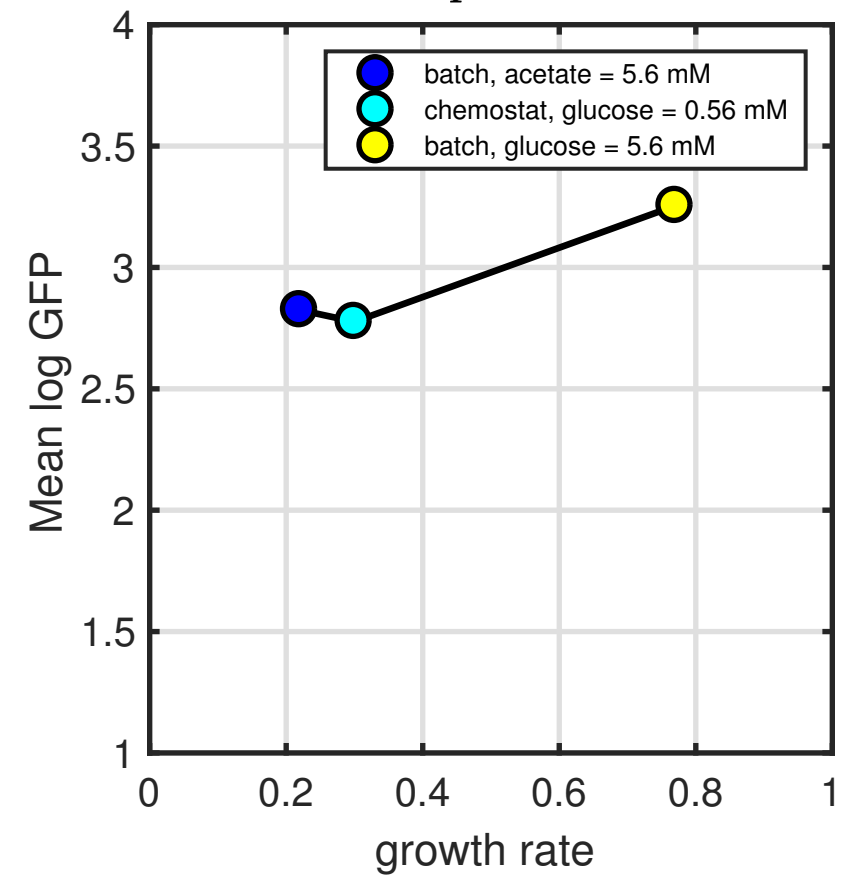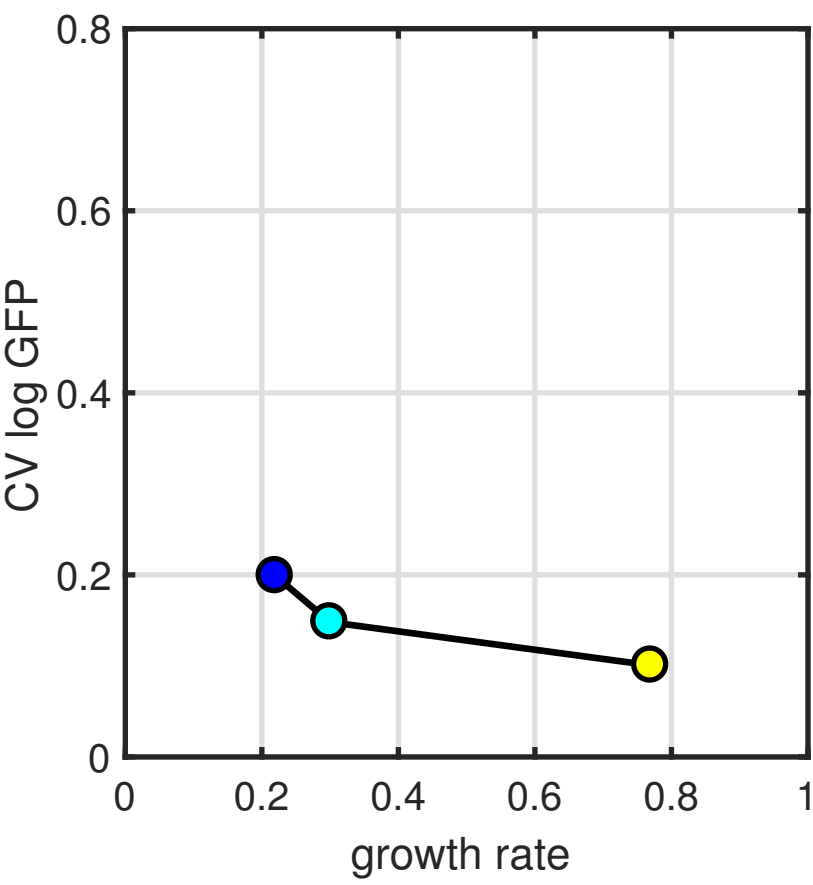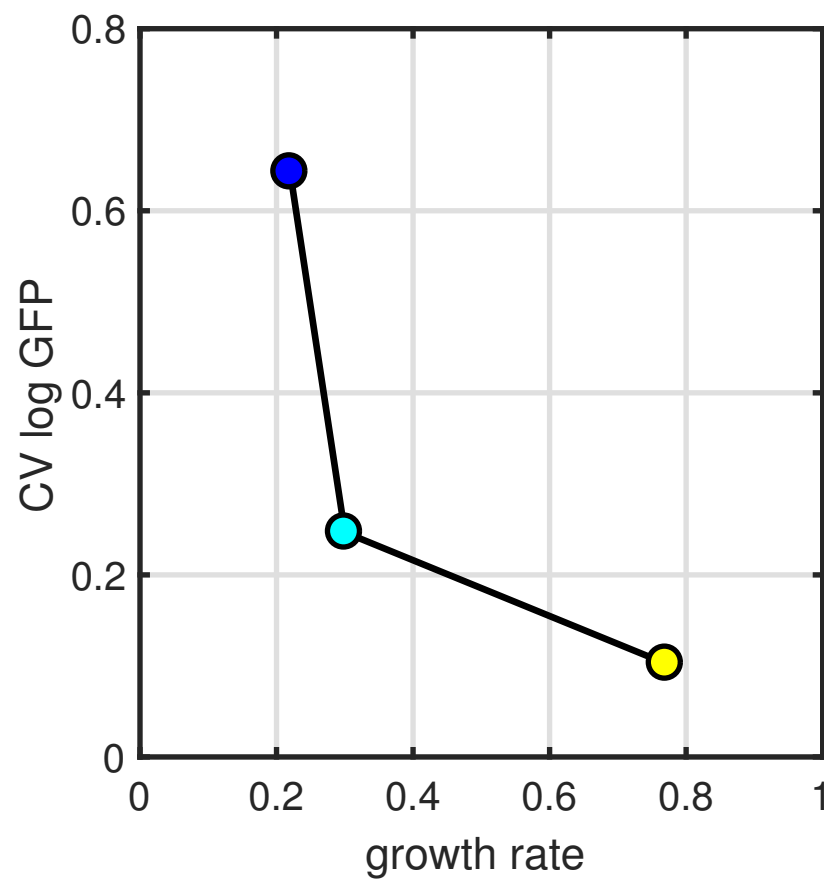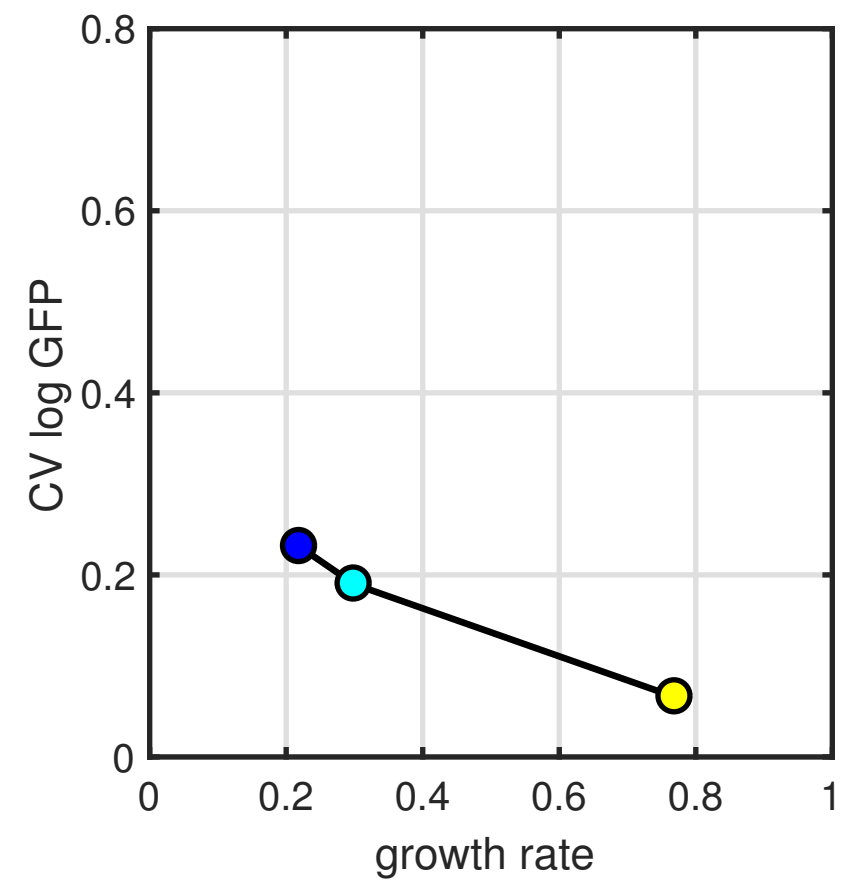

Supplement: Supplemental Figure 6: Single-cell expression data at different growth rates [file rsos172234supp7.pdf]

**CV protein number (at birth)**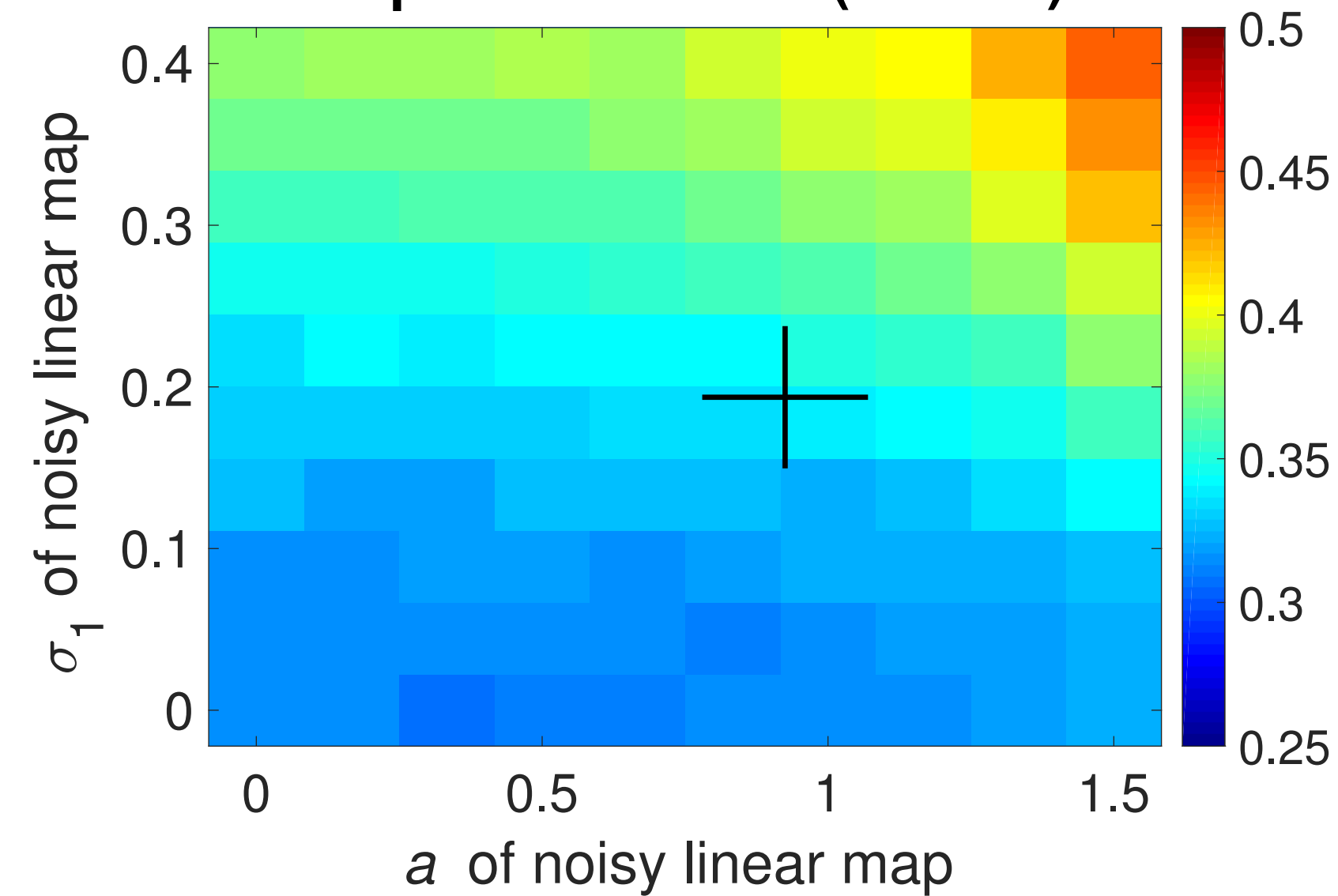**CV protein concentration (at birth)**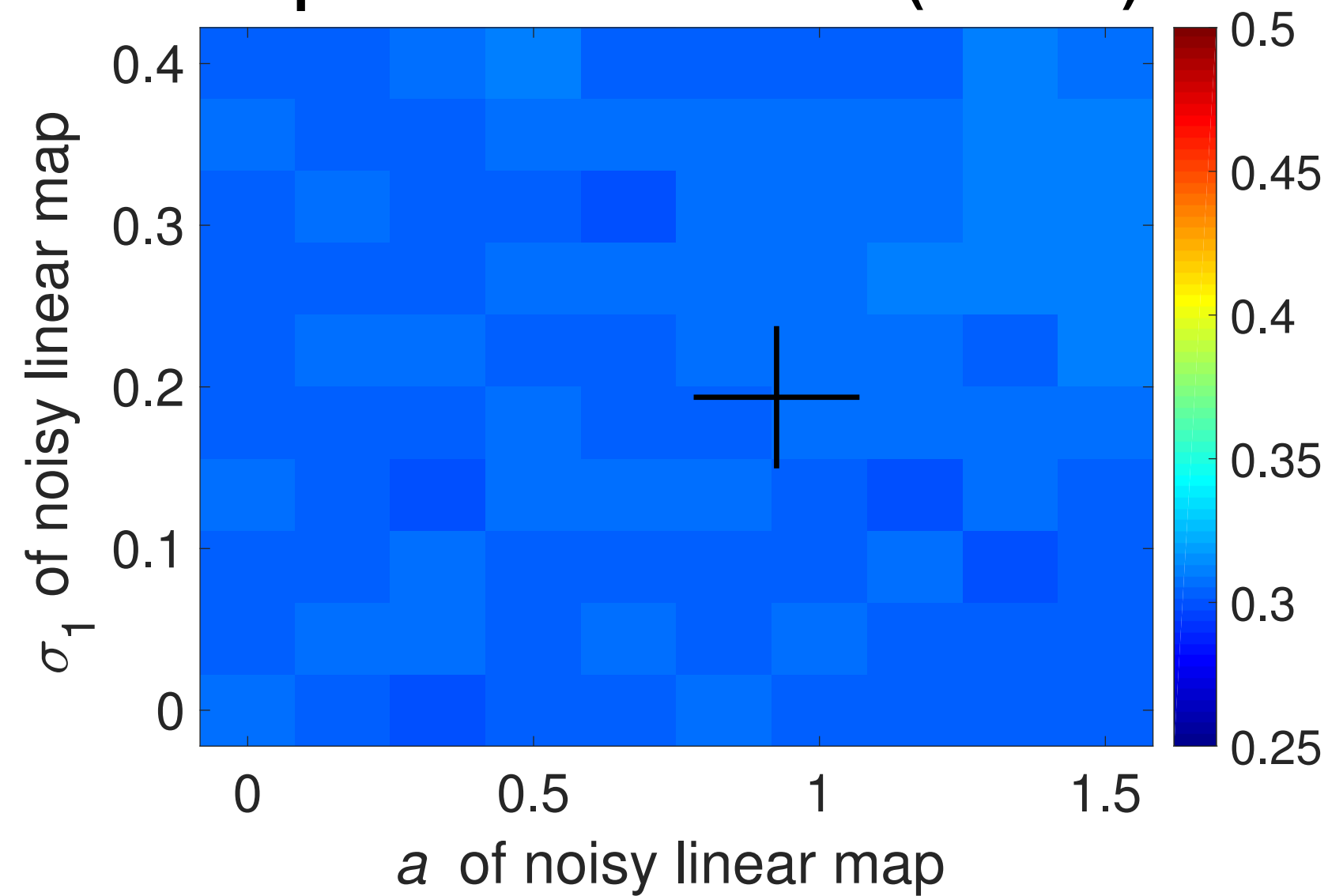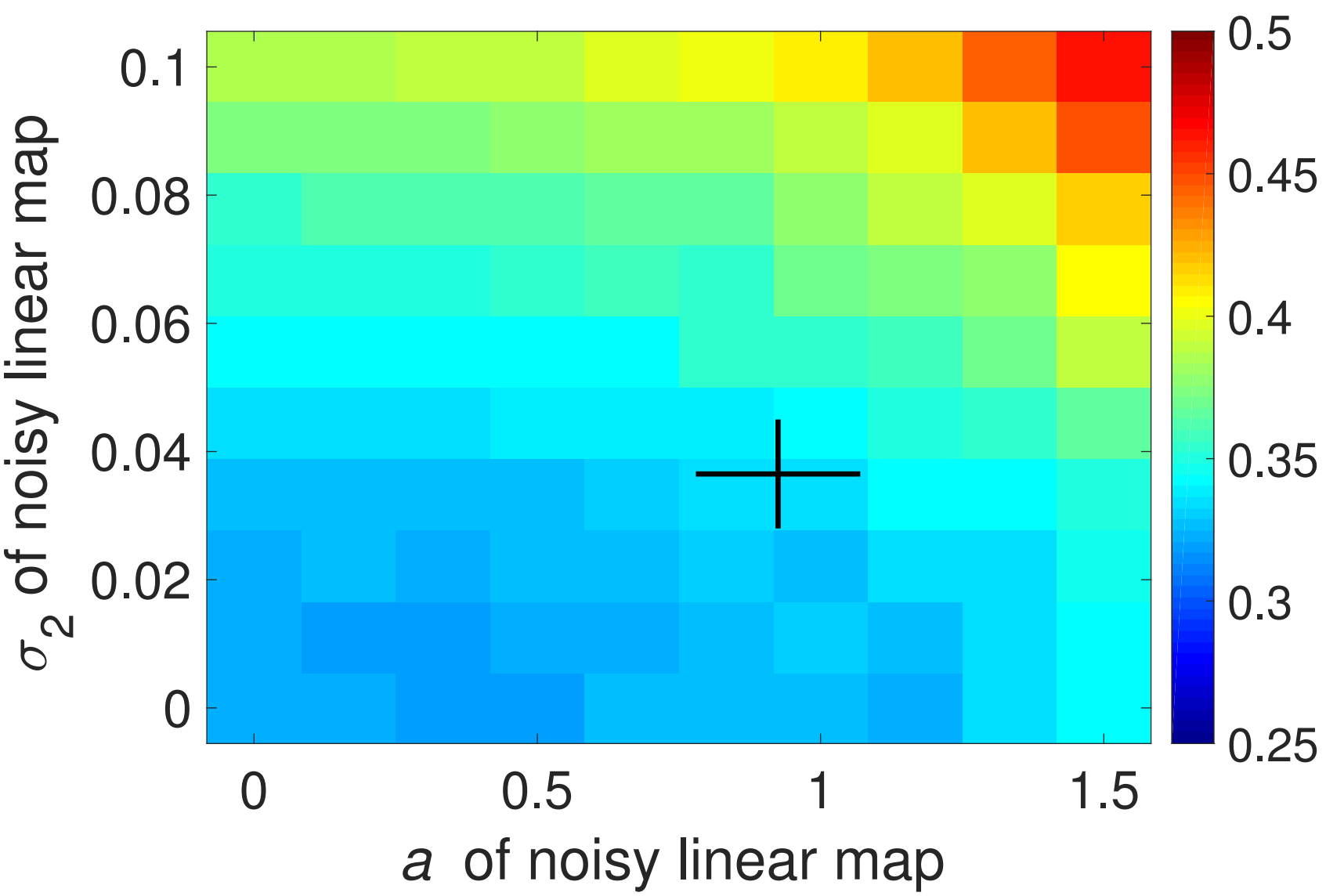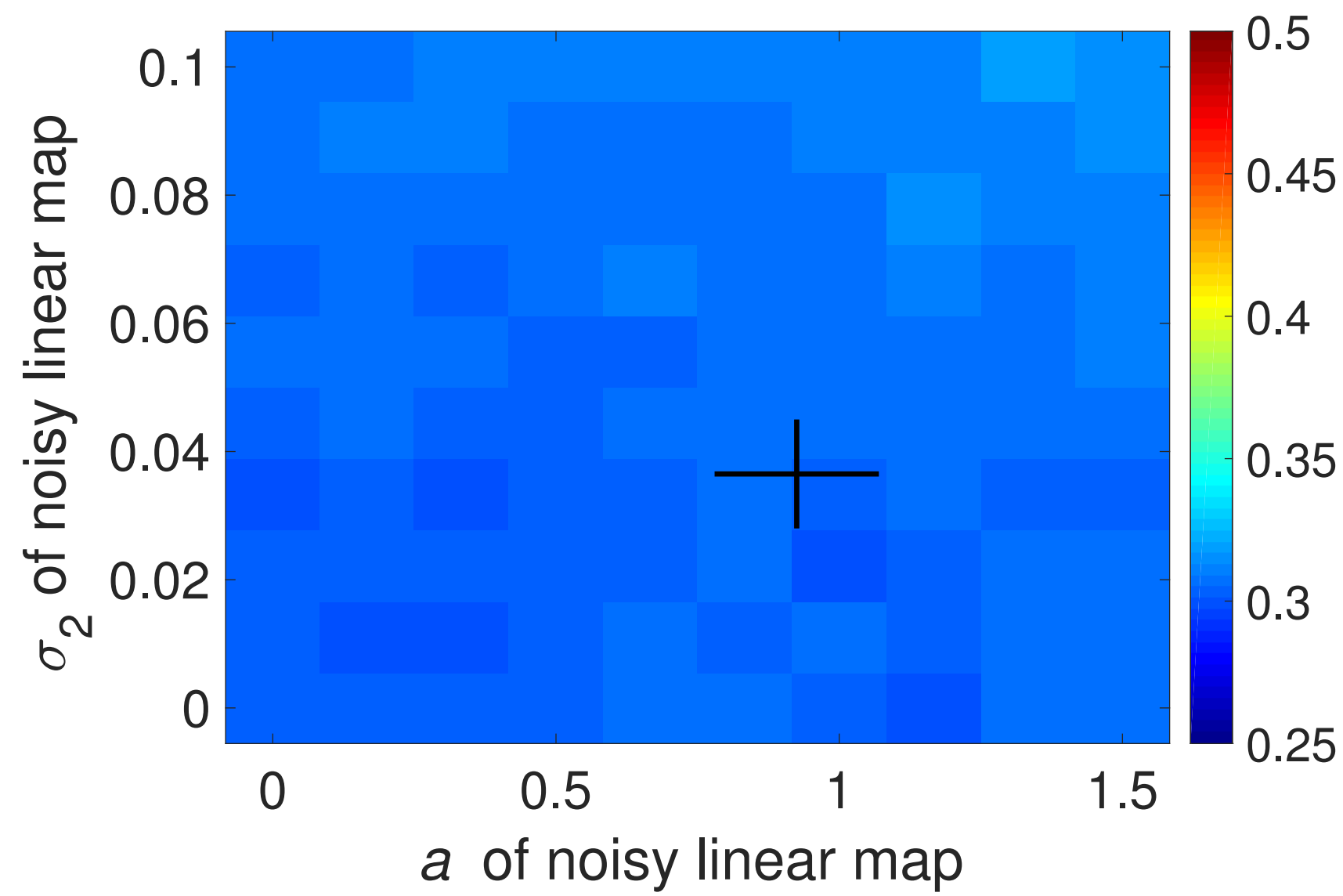

Supplement: Supplemental Figure 7: Protein concentration noise is robust to variability in size when the transcription rate scales with cell size [file rsos172234supp8.pdf]
